# Fungal Contaminants in Drinking Water Regulation? A Tale of Ecology, Exposure, Purification and Clinical Relevance

**DOI:** 10.3390/ijerph14060636

**Published:** 2017-06-13

**Authors:** Monika Novak Babič, Nina Gunde-Cimerman, Márta Vargha, Zsófia Tischner, Donát Magyar, Cristina Veríssimo, Raquel Sabino, Carla Viegas, Wieland Meyer, João Brandão

**Affiliations:** 1 Department of Biology, Biotechnical Faculty, University of Ljubljana, Jamnikarjeva 101, 1000 Ljubljana, Slovenia; nina.gunde-cimerman@bf.uni-lj.si; 2 Department of Water Hygiene, National Public Health Center, Albert Flórián út 2-6, H-1097 Budapest, Hungary; vargha.marta@oki.antsz.hu; 3 Department of Biology, University of Veterinary Medicine, István utca 2, H-1078 Budapest, Hungary; zsofi.tischner@gmail.com; 4 Department of Air Hygiene and Aerobiology, National Public Health Center, Albert Flórián út 2-6, H-1097 Budapest, Hungary; magyar.donat@gmail.com; 5 Department of Infectious Diseases, National Institute of Health Doutor Ricardo Jorge, Av. Padre Cruz, 1649-016 Lisboa, Portugal; cristina.verissimo@insa.min-saude.pt (C.V.); raquel.sabino@insa.min-saude.pt (R.S.); 6 GIAS, ESTeSL—Escola Superior de Tecnologia da Saúde de Lisboa, Instituto Politécnico de Lisboa, 1990-096 Lisbon, Portugal; carla.viegas@estesl.ipl.pt; 7 Molecular Mycology Research Laboratory, Centre for Infectious Disease and Microbiology, Sydney Medical School, Westmead Hospital, Marie Bashir Institute for Emerging Infectious Diseases and Biosecurity, Westmead Institute for Medical Research, The University of Sydney, Level 4, Room 0.4.04, 176 Hawkesbury Road, Westmead, NSW 2145, Australia; wieland.meyer@sydney.edu.au; 8 Department of Environmental Health, National Institute of Health Doutor Ricardo Jorge, Av. Padre Cruz, 1649-016 Lisboa, Portugal

**Keywords:** drinking water, fungi, fungal contaminants, *Aspergillus*, in water, Candida, moulds, molds, mycotoxins

## Abstract

Microbiological drinking water safety is traditionally monitored mainly by bacterial parameters that indicate faecal contamination. These parameters correlate with gastro-intestinal illness, despite the fact that viral agents, resulting from faecal contamination, are usually the cause. This leaves behind microbes that can cause illness other than gastro-intestinal and several emerging pathogens, disregarding non-endemic microbial contaminants and those with recent pathogenic activity reported. This white paper focuses on one group of contaminants known to cause allergies, opportunistic infections and intoxications: Fungi. It presents a review on their occurrence, ecology and physiology. Additionally, factors contributing to their presence in water distribution systems, as well as their effect on water quality are discussed. Presence of opportunistic and pathogenic fungi in drinking water can pose a health risk to consumers due to daily contact with water, via several exposure points, such as drinking and showering. The clinical relevance and influence on human health of the most common fungal contaminants in drinking water is discussed. Our goal with this paper is to place fungal contaminants on the roadmap of evidence based and emerging threats for drinking water quality safety regulations.

## 1. Introduction

Fungi are ubiquitous, heterotrophic organisms present in oceans, fresh water and drinking water. They can be divided based on the ability to colonize different environments into three groups: as mesophilic fungi, generalists and specialists [1,2]. Mesophilic species inhabit niches with moderate physicochemical parameters, while generalists grow under changing life conditions, but with growth optimum under moderate conditions. Specialists inhabit extreme habitats and are unable to grow under moderate conditions [1]. Ecologically, fungi are saprophytes, degrading organic matter, with some species acting also as parasites or symbionts [3,4]. Due to their diverse life cycle, ability to form large hyphal networks and produce spores, or growing as single yeast-cells, they maximize nutrients uptake and can survive under various life conditions, one of them being oligotrophic water systems [2]. In the last 30 years, the presence of a high variety of fungi was reported from European water, including surface-, ground- and tap water intended for human consumption [2]. It is thus imperative that we regard fungi as nature’s resilient recycling machines, when we supply drinking water to users who may lack standard natural abilities to fight back. 

Using cultivation techniques, ascomycetous filamentous fungi were those mainly detected, classified as members of the genera *Acremonium*, *Alternaria*, *Aspergillus*, *Cladosporium*, *Fusarium*, *Penicillium* and *Trichoderma*. The second most cultivated group were fungi from the subphylum Mucormycotina (former phylum Zygomycota) [5,6,7,8,9,10,11,12,13,14,15,16,17,18,19]. The presence of yeasts from surface-, ground- and tap water was rarely reported, probably due to the cultivation bias [19]. Numbers and diversity of fungi were reported to be higher in surface water in comparison to ground- and tap water; environmental factors, such as high contents of organic nutrients, varying temperature, pH, and water flow being the main reason why [15,20,21]. During the production of tap water, cleaning processes including techniques for removing large particles from raw water, and addition of chlorine contribute to a lower load of fungi. Yet, some species remain present in tap water, later establishing biofilms that persist in water distribution systems [22,23]. Reservoirs before elevation stations, positive pressures in building distribution designs, preventive maintenance, permanent running water in the system and adequate residual disinfectant are examples of how the distribution system should be operating [24,25].

Presence of fungi in biofilms and their interactions with other microorganisms remain poorly understood, even though in recent years the use of metagenomic approaches brought more detailed insight to this field [23,26,27]. Fungi growing in biofilms inside taps and in tap water affect the taste and odour, interfering with the chlorination process, due to the release of a large scale of products known as secondary metabolites. These may be very diverse and specific for different fungal species [28]. While the role of secondary metabolites in the ecology of fungi is to defend their habitat, and suppress the growth of competitors [29], some of them are toxic to animals, and may present a risk for human health in higher concentrations or under prolonged time of exposure [30]. Not only secondary metabolites, but also fungal cell wall components and the fungal load itself may contribute to the emergence of allergies and other opportunistic and systemic infections, mainly in immunocompromised individuals [31,32]. Although in the last few decades fungi are becoming frequently recognized as causative agents of respiratory, mucosal, rhinocerebral, cutaneous and subcutaneous infections [32], they remain largely overlooked in the regulations of water quality and consumption [2]. Possible reasons may be the lack of knowledge of the fungal load in water, divergent cultivation methods, heterogeneous mechanisms of fungal pathogenicity and consequently the low number of reports connecting fungal presence in tap water and the occurrence of diseases in humans [21]. Also, unlike obvious outbreaks, low prevalence afflictions are handled discretely, and rarely explored as to how they originate. 

The present paper represents a joint review on the presence of fungi in surface water, groundwater and tap water from European countries reported in the last 30 years. It includes known ecological and anthropogenic factors contributing to the presence of fungi in water, together with the mostly used methods for their cultivation and detection, followed by a sustained clarification of the possible relevance of these organisms in drinking water and a recommendation concurred by the authoring team.

## 2. Fungi and Water—Background Information

### 2.1. Regulations

Though the presence of fungi in water distribution system and the associated health risks are well documented in the scientific literature, inclusion of fungi in the drinking water regulations is scarce. Most national and international guideline documents (including the World Health Organization) list fungi among the “nuisance organisms” causing odour problems, and do not deem dedicated monitoring necessary [33,34]. The U.S. EPA considered the inclusion of microsporidia in drinking water regulations earlier, but it was withdrawn from the list of “Contaminant Candidate List” in a later phase [35,36]. The European Union drinking water directive does not address fungi explicitly either. However, the directive states that wholesome drinking water should be “free from any micro-organisms and parasites and from any substances which, in numbers or concentrations, constitute a potential danger to human health” [37]. This definition implies that the presence of pathogenic or allergenic fungi in the drinking water is not acceptable either. The obligatory microbial drinking water parameters (*E. coli*, *Enterococci*, coliforms or clostridia) have no indicative value of fungal contamination. The indicator parameter heterotrophic plate count (HPC), however, may include fungi as well. HPC is widely used to indicate changes in microbial concentration (i.e., ingress or regrowth in the drinking water distribution system [38]. Regulatory value is generally not rendered to HPC. The EU directive does not give a parametric value; compliance is defined as “no abnormal change”. 

Only a limited number of member states have additional, more specific regulation. The Czech drinking water legislation requires light microscopic analysis of drinking water samples concentrated by centrifugation. It gives a collective parametric value of 50 individuals/mL for all “microscopic organisms” [39] including all eukaryotes and cyanobacteria, which are visible under the microscope. Analysis extends to the visual identification of the observed microorganisms, e.g., the filaments and spores of micromycetes. The Hungarian drinking water act takes a similar approach. Samples are concentrated by membrane filtration and analysed by light microscopy. However, parametric values are given by groups of organisms separately (for fungi, 0 individuals/L) [40]. The Swedish legislation is the only one that requires the direct detection of fungi by culture. It lists “microfungi” (including moulds and yeasts) as an indicator parameter, with a parametric value of 100 CFU/100 mL [41]. All three of the above requirements apply for drinking water samples at the point of compliance (i.e., the consumer’s tap). National standards are used for detection and enumeration (CSN 75 7712, MSZ 448-36:1985 and SS 028192, respectively). 

### 2.2. Ecology of Fungi in Water

Fresh water available for human consumption represents only 0.6% of global water supplies stored in glaciers, running surface water and groundwater [4]. Depending on geological features of the area, either groundwater or surface water is used as a primary source to produce tap water [2,42]. In other regions of the world, rainwater is also a relevant source. Therefore, the presence, colonization and growth of fungi in tap water depends on several factors, such as location of primary water source, sun irradiation, temperature, ion composition and pH, presence of organic material, dissolved oxygen concentration, water treatment, use of materials for water distribution systems and consequently the possibility of biofilm formation [2,4,12,19,43,44,45,46]. 

### 2.3. Aqueous Geochemistry Processes Affect the Presence of Fungi in Water and Vice-Versa

Locations of aquifers and primary water sources are naturally determined by geological features, not only influencing water availability from the main water bodies, but also their physico-chemical properties [4,19]. Water in predominantly rocky areas, with low solubility, have less diverse ion composition, and are more likely present on the surface or as a groundwater close to the surface [47]. On the other hand, geological structures, such as limestone composed from calcium carbonate, have a significant effect on the formation of specific areas, known as karst systems [48]. Water in such areas dissolves the ground faster, thus water bodies are frequently absent from the surface and are more likely present in form of carbonate-rich groundwater inside the cave systems [47,48]. Chemical properties of water influence fungal presence in water systems, and vice-versa. Fungi were proven to be actively involved in aqueous geochemistry processes, such as dissolution and corrosion of rocks and precipitation of minerals [46,48]. In general, rocks with alkaline pH proved to be more susceptible to fungal colonization than rocks with acidic pH [49]. Besides limestone also the presence of other rock types, such as andesite, amphibolite, basalt, dolerite, gneiss, granite, marble, sandstone, soapstone and quartz, positively influence the growth of fungi, like *Aschersonia* spp., *Aspergillus niger*, *Penicillium expansum*, *P. simplicissimum*, *Scopulariopsis brevicaulis*, and a wide range of melanized, meristematic fungi, known under the umbrella-term “black yeasts” [48,50,51]. The latter include species of the genera *Aureobasidium*, *Exophiala*, *Phaeotheca* and *Trimmatostroma*, and were globally isolated from different rocks exposed to sun irradiation, salty and fresh water, and from statues of cultural heritage in urban cities [51]. Fungi are influencing biological weathering of rocks and together with chemical weathering they are contributing to changes in pH and ion composition of water [50]. 

The pH of water has shown to have an important role on fungal presence, their growth and bioremediation processes. Positive correlation was observed between the growth of aquatic hyphomycetes and pH between 5 and 7 [20,52], and confirmed recently in a study of deep groundwater reporting the highest diversity in mixed fungal communities at slightly lower pH [47]. Acidic pH has a positive influence on binding of heavy metals like manganese and cadmium to the fungal cell wall components [53], which can be beneficial for some fungal species. For instance, species of plant- and water-related fungi *Paraconiothyrium* and *Phoma* stabilize and oxidize manganese ions by organic acids and use them in degradation of phenolic structures [54]. Metal-binding onto or around fungal hyphae, under acidic conditions, represents sink for heavy metals (e.g., aluminium, copper and zinc) in environment and high bioremediation potential of aquatic fungi [50,55]. Changes in pH in the environment are related also with the polymorphic growth of certain fungi, with low pH inducing growth of round, swollen hyphal cells or yeast-like cells, as observed for *Alternaria*, *Fusarium* and *Mucor* species [52,56,57]. Some species of black yeasts, like *Exophiala dermatitidis* were reported to form thick cell walled muriform clumps [56,58]. Changes in growth form lower the pH-induced stress allowing fungi a more efficient intake of nutrients and the survival under extreme conditions. The pH-induced stress could be additionally lowered with the intake of certain ions, like calcium. This has been shown for *E. dermatitidis* [56,57]. A recent study conducted by Novak Babič et al. [19] showed a positive correlation between higher concentrations of calcium and magnesium ions, contributing to the water hardness, and the presence of fungi in water [19]. Not only inorganic ions, also carbon availability, nitrate, phosphate and sulphate positively correlated with the presence and diversity of fungi in water systems; suggesting an important role of fungi in geochemical cycles of metals, carbon, nitrogen and sulphur in water habitats [4,19,46,47,50]. Additionally, the presence of nitrate and phosphate in water has been shown to be important for fungal growth and the effective breakdown of long-chained components of plant material and other organic matter [59].

### 2.4. Number and Diversity of Fungi Depends on Organic Matter Originating from Natural and Anthropogenic Sources

The concentration of organic matter in water depends on the location and the surface area of water bodies [4,43,44,45]. Small surface water bodies or water with low flow receive the most of organic matter due to the plant vegetation, and larger water bodies and streams on high altitude are mainly supplied with organic matter due to the algal primary producers [4]. Surface water with slow flow close to the stream mouth are rich on nitrate, nitrite, phosphate and other products of organic material degradation, such as plant debris, lignin, hemicelluloses and cellulose [4,60]. Besides these, also human habitation may contribute to the water pollution with organic substances via fertilizers or industrial and household waste [61,62]. Consequently, surface water contains high biomass and rich diversity of plant degrading filamentous fungi [63]. In Europe, the majority of the isolated fungal species from surface-, ground- and tap water belong to the ascomycetous genera *Alternaria*, *Aspergillus*, *Cladosporium*, *Fusarium*, *Gibberella*, *Penicillium*, *Phoma*, *Sarocladium*, *Scopulariopsis*, *Sporothrix*, *Talaromyces* and *Trichoderma*, but also fungi from subphylum Mucormycotina, such as *Absidia*, *Mortierella*, *Mucor*, *Rhizopus* and *Umbelopsis* were regularly isolated (Table 1). The presence of yeasts has been reported sporadically. Reports have been limited mainly to the genera of basidiomycetous yeasts *Cystobasidium*, *Naganishia* (former *Cryptococcus*) and *Rhodotorula* (Table 1) [8,20,64]. The presence of the human pathogen *Candida albicans* (Ascomycota) in surface water has been reported only once [17]. Among black yeast-like fungi only the plant-related species *Aureobasidium pullulans* has been isolated directly from surface water [20], while *Cyphellophora catalaunica*, *Exophiala aquamarina*, *E. lacus*, *E. oligosperma*, and *Rhinocladiella similis* were associated with river sediments [65].

In comparison to surface water, groundwater contains more inorganic ions, but usually lacks organic nutrients provided by plants and algae. Low amounts of organic nutrients are present mainly in the form of mono- or polysaccharides derived from the remains of bacterial biofilms [47,50]. Thus, the presence of fungi in groundwater associated with degradation of plant debris is limited or reported less often. On the other hand, oligotrophic conditions support growth of melanised fungi, such as *Aureobasidium melanogenum*, high diversity of *Exophiala* species and *Rhinocladiella similis* (Table 1) [9,11,19,23,26,66,67]. These species were regularly reported from different European countries from both ground- and tap water, but were rarely reported in a relation to surface water, pointing toward groundwater as the main source of contamination of tap water with these opportunistic pathogenic fungi (Table 1) [19]. 

Environmental water in areas with dense human population do not only contain high amounts of organic waste, but contain compounds of anthropogenic origin, such as organohalogens, pesticides, xenobiotics and long-chained aromatic hydrocarbons (benzene, toluene, ethylbenzene and xylene, known as BTEX) [68]. The later derive from crude oil and fuels, and are released in the environment by partial combustion of coal and other fuels, or accidental spills [68,69]. Although their presence may be toxic for most organisms, certain fungi assimilate them as a sole source of carbon [70,71]. Breaking down long-chained pollutants is a well documented feature of the black yeasts *Aureobasidium pullulans*, *Cladophialophora* spp., *Exophiala dermatitidis*, *E. jeanselmei*, *E. mesophila*, *E. oligosperma*, *E. xenobiotica*, *Graphium* sp., and *Rhinocladiella similis* [68]. Table 1 displayes also a wide range of filamentous fungi from the genera *Acremonium*, *Alternaria*, *Aspergillus*, *Beauveria*, *Chrysosporium*, *Cladosporium*, *Fusarium*, *Geomyces*, *Geotrichum*, *Gliocladium*, *Graphium*, *Paecilomyces*, *Penicillium*, *Scedosporium*, *Scopulariopsis*, *Sepedonium*, *Stachybotrys*, *Trichoderma*, and *Verticillium* [4,52,72,73] that exhibit the same ability (and have been detected in both, surface- and groundwater).

Particularly in closed surface water bodies with low flow rates the high concentration of organic nutrients and pollutants leads to an overgrowth of algae and bacteria, lowering the amount of oxygen [4]. Oxygen concentration decreases also with the depth in both, surface- and groundwater [52]. Since fungi are in general aerobic microorganisms, depletion of oxygen can negatively affect fungal biomass production in water systems with low oxygen concentrations [45]. However, some fungi do not only sustain the lack of oxygen, but also grow under anaerobic conditions by adaptation of their metabolism and growth form [4,74,75]. Species from the genera *Aspergillus*, *Nectria*, *Fusarium* and *Penicillium* growing as facultative anaerobes, using nitrate or nitrite as alternative terminal electron acceptors in the absence of oxygen, falling under this category [76,77]. Some *Mucor* species, for example, grow in hyphal networks in the presence of oxygen, but change to a yeast-like form under anaerobic conditions [78]. Similar situations were observed for species from the genera *Aureobasidium* and *Candida* [4,52]. Besides these, another important adaptation at low level of water and oxygen is the formation of buoyant conidia occurring in many water-related fungal species [4].

### 2.5. Effect of Sunlight and Water Temperature on Fungi in the Natural Environment

Not only chemical processes, but also physical factors contribute to fungal presence in raw water sources. The most important may be the effect of sun irradiation and consequently changes in the water temperature. The effect of sunlight irradiation is stronger in high altitude areas and in low flow surface water [2]. It consists of infra-red, ultra-violet (UV) and visible spectre of the light; among those, the effect of the UV-radiation causes the highest damage of cell mechanisms and is thus the most studied [90]. Natural solar disinfection is a proven technique for generating safer drinking water, particularly by inactivation of faecal bacteria [91,92]. However, the effect on fungi is not well documented. Tests with simulated solar disinfection successfully lowered the number of the species *Alternaria alternata*, *Fusarium equiseti*, *F. oxysporum*, *F. solani*, *F. verticillioides* and *Candida albicans* in water samples [92,93,94,95], while fungi with melanised cell walls were less susceptible [2]. The effect of solar UV-radiation varies with the time of the day, is lower during cloudy days, in large volumes of water, and in water with high contents of organic matter with increased turbidity [95,96]. Together with the DNA-damaging effect of UV-radiation, solar disinfection contributes also to the thermal disinfection with raising the water temperature [92]. The water temperature depends also on the depth, volume, and flow rate (higher effect in shallow waters with low flow rates) [95]. Normally, temperatures of running surface water in temperate climate are below optimal growth temperatures of most water-related fungi, with growth peaks between 15 °C and 25 °C, but may vary over the seasons [97]. Also the structure of fungal communities in surface water is not stable [52], with a higher content of thermotolerant *Aspergillus* and *Phialophora* species and yeasts [11] during the summer, being replaced by filamentous fungi from the genera *Acremonium*, *Cladosporium* and *Penicillium* during the cold seasons [13,98,99].

**Figure 1:**
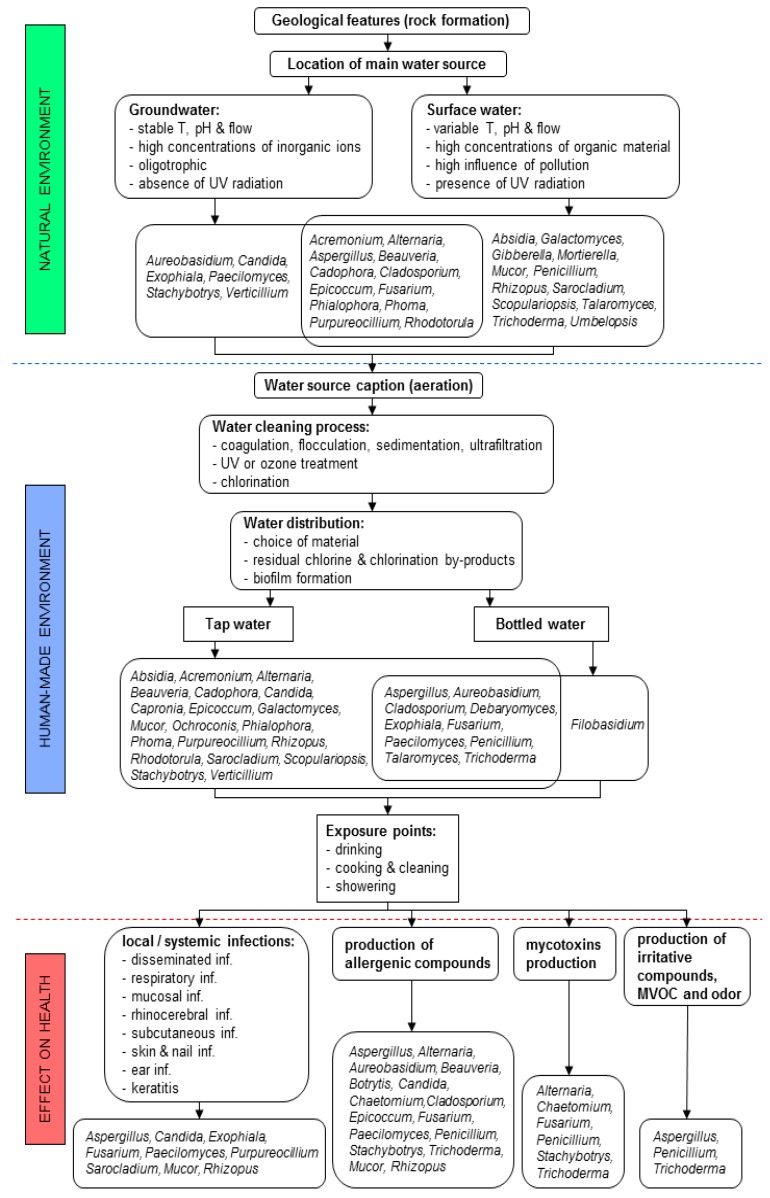
Abiotic, biotic and anthropogenic factors influencing fungal presence in groundwater, surface water, tap water and non-mineral bottled water, with possible effect of fungi on human health via different exposure points. The most common factors having an influence on the fungal presence and diversity in different water sources divided into factors influencing fungal presence, mainly in raw water sources in the natural environment (indicated with green colour), anthropogenic factors influencing fungal presence during production of tap and non-mineral bottled water, and exposure points of fungi via water-related activities (indicated with blue colour). Red colour indicates the most frequently detected fungal genera from tap and bottled water with their possible effects on human health.

Abiotic and biotic conditions in natural water habitats play an important role for the presence and diversity of fungi. Although being still largely unexplored, the above-described factors have an influence on the water quality in natural environments and as such, they need to be taken into consideration during the processes of tap water production (Figure 1).

### 2.6. Effect of Drinking Water Treatment Processes on Fungal Contaminants

Until the end of the 19th century, water for human consumption was derived to the public either from groundwater, or rivers and springs upstream of habitation [42]. With the concentration of growing populations in large areas and cities, supplying clean water became a problem, resulting in major cholera outbreaks in Europe [42]. After the expanding knowledge in microbiology, contaminated water became connected with water-borne and faecal-borne diseases, and the first water treatment practices (first mechanical sand filtration, then coagulation-sedimentation processes) were implemented [42]. Shortly after, Robert Koch showed for the first time that chlorine is effective against *Vibrio cholerae* and other waterborne bacteria [100]. Today, the water industry is using a combination of techniques to provide pathogen-free drinking water (Figure 1). Chlorine, introduced with the beginning of the 20th century, is still the most common disinfectant [42]. 

The first step in the process of raw water purification starts with aeration in reservoirs for the removal of volatile compounds and gases from raw water sources [22]. The most commonly used technique is cascade aeration. During the process, air is blown and mixed into the water [22]. An alternative technique is the use of compressed air, introduced into water through a system of perforated pipes, which is generally used for the removal of iron and manganese [22]. However, air based treatment steps are one of the possible contamination sources by airborne fungal particles. The nest step is usually coagulation of the suspended particles by adding chemical agents (coagulants) [22]. After adding coagulants both the visible particles and microorganisms combine into larger flocks, which sediment and are then removed by filtration [22]. The process usually removes cysts of protozoa (e.g., *Giardia* spp.), as well as most other microorganisms and some viruses [101]. The most commonly used coagulants are aluminium and iron salts (aluminium sulphate, ferric sulphate, ferric chloride), which act primarily by changing the pH of water to less alkaline values. They may be used together with positively charged polymers, or alternatively be replaced by negatively charged organic polymers, often used in a combination with metal coagulants [102]. Larger flocks sediment whereas smaller flocks are removed by filtration, with cellulose, sand, charcoal or fabrics filters [22,103]. Primary filtration may be replaced or followed by ultrafiltration or microfiltration [22]. The process can be combined with active carbon for the adsorption and removal of dissolved small organic molecules, such as trihalomethanes and pesticides [22,103]. These methods have different effects on microorganisms, and can be used against them with different degrees of efficiency. Data available generally cover various microorganisms causing enteric diseases but no fungi. Coagulation, flocculation and sedimentation may remove approximately 30% of bacteria, 30–70% of viruses and 30–99.99% of protozoa. The efficacy depends on the coagulants used, pH, temperature and turbidity of water [22]. Efficacy of filtration depends on the pre-treatment and the used membranes, thus the removal may vary between 30% and 99.99% for bacteria, 50–99.99% for protozoa and 20–99% for viruses [22]. The WHO does not report any values for fungi, however, it has been shown that sand filtration may remove between 8% and 90% of fungi, coagulation process 54%, and the sedimentation process 70% [83,104]; none remove 100%. Not all treatment steps are used always; the quality of the saource water will determine the process. 

Water after filtration is usually still not suitable for human consumption, thus additional disinfection is needed. Disinfection is, depending on the site of action, divided into primary and secondary. Primary disinfection destroys microorganisms in the raw water stored in reservoirs. Secondary or residual disinfection inhibits the growth of microorganisms in the water supply network [105]. The choice of disinfection methods depends on the water quality after treatment, availability of materials and cost. UV-radiation is commonly used in smaller facilities [2,22]. UV disinfection is carried out without addition of any substances to the water, and therefore does not leave toxic by-products. Its biocidal effect is reached between 180 nm and 320 nm and is also highly dependent on the water turbidity (dissolved organic particles), water flow, and on pigmentation of the cells and spores [2,22,106,107]. According to WHO a 99% reduction may be achieved under a dosage of 7 mJ/cm^2^ for bacteria, between 5 mJ/cm^2^ and 10 mJ/cm^2^ for protozoa and 59 mJ/cm^2^ for viruses [22]. A fungicidal effect on single strains of yeasts, such as *Candida albicans*, *C. glabrata*, *C. krusei*, *C. parapsilosis* and *C. tropicalis*, was achieved after 10–45 min at the wavelength of 254 nm. To achieve the effect with the same wavelength for filamentous fungi, such as *Aspergillus fumigatus*, *A. niger*, *Microsporum canis* and *Trichophyton rubrum*, 75 min of exposure were required [90,106]. 

Primary disinfection of water may also be achieved also with the ozonation. Ozone, as a strong oxidizing agent has many advantages, such as oxidation of inorganic and organic chemicals increasing their biodegradability and removing the colour, smell and taste from water [2,22]. Under proper dosage and contact time it does not leave any by-products, though under some conditions, mutagenic and carcinogenic by-products may be generated (e.g., bromate) [108]. Ozone-enriched air is introduced directly into water in contractor tanks, providing between 10 min and 20 min of contact time [22]. Effect of ozonation against viruses, bacteria and protozoa is better at slightly acidic pH (6–7) and temperatures between 15 °C and 20 °C [22]. Ozonation proved to be effective against different fungi and their spores. Tested species included single strains of *Aspergillus brasiliensis*, *A. flavus*, *A. fumigatus*, *A. niger*, *Candida albicans*, *C. parapsilosis* and *Fusarium oxysporum* complex [109,110,111,112,113,114,115]. Although used as an alternative for chemical disinfection, UV and ozone disinfection do not provide residual effect and are usually combined with a chlorination process.

Chlorination is used for primary and secondary microbial disinfection of water. The most widely used forms of chlorine for water disinfection are chlorine gas or hypochlorite in the form of powder as calcium hypochlorite (Ca(OCl)_2_) or as liquid sodium hypochlorite (NaOCl). Both are suitable for the disinfection of water with a low content of organic substances. Chlorine dioxide is used when better penetration into the biofilms formed on the walls of pipelines and tanks is needed [42,116]. Optimal disinfection with chlorine and its derivatives is usually achieved at temperatures between 15–20 °C and pH between 7.0 and 7.5. Additionally, water should contain the least possible amount organic material, iron, manganese and ammonia, due to chlorine reactions with these agents, lowering its residual effect [22,42]. The free chlorine concentration in chlorination tanks must reach >0.5 ppm, with the contact time being at least 30 min to inactivate bacteria and protozoa [42]. For the proper residual effect, final concentrations of free chlorine in the water supply network must be between 0.3 mg/L and 0.5 mg/L [42]. During the chlorination process, aqueous chlorine reacts with ammonia and forms chloramines. These exist in the form of mono-, di- and trichloramines, but only monochloramine has useful disinfection effect. Although it is less effective against microbes than free chlorine, it is persistent and provides a stable residual effect through the water supply network [22,42]. While both free chlorine and monochloramine have a known effect on viruses, bacteria and protozoa [22], little is known about their effect on fungi. A variety of fungal species belonging to the genera *Acremonium*, *Alternaria*, *Aspergillus*, *Aureobasidium*, *Beauveria*, *Botrytis*, *Candida*, *Chaetomium*, *Cladosporium*, *Epicoccum*, *Exophiala*, *Fusarium*, *Geotrichum*, *Gliocladium*, *Mortierella*, *Mucor*, *Naganishia*, *Ochroconis*, *Paecilomyces*, *Penicillium*, *Phoma*, *Rhizopus*, *Rhodotorula*, *Sarocladium*, *Sporotrichum*, *Sporothrix*, *Stachybotrys* and *Trichoderma* have been cultivated from chlorinated water, pointing out possible resistance to the regular chlorination process (Table 1) [2]. However, tested free-chlorine concentrations between 1 ppm and 2 ppm in 97–99% inactivated single strains of *Trichoderma harzianum*, *Epicoccum nigrum* and *Aspergillus niger* after the exposure time of 60, 40 and 10 min, respectively [117]. A recent study, conducted by Pereira et al. [118] showed that single strains of the filamentous fungi *Aspergillus fumigatus*, *A. terreus*, *Cladosporium cladosporioides*, *C. tenuissimum*, *Penicillium citrinum*, *P. griseofulvum* and *Phoma glomerata* were more resistant to chlorination than viruses and bacteria and less resistant than protozoan oocysts. The study also confirmed slightly acidic pH and temperatures ~20 °C as the best chlorination conditions for fungal inactivation [118].

### 2.7. Materials Used for Building Water Supply Networks and Their Effect on Biofilm Formation

Following chemical disinfection, the quality of water is checked, and if suitable for drinking, it is delivered to consumers via water supply networks. The network pipe systems are built of different materials and they may interact with residual chlorine and chlorination by-products. They may influence microbiological quality of water as well, due to possible biofilm formation [2]. The European Union (EU) does not have a unified approach for materials and products in contact with drinking water. Thus, in 2011, four member states (4 MS; France, Germany, The Netherlands and the United Kingdom) standardized procedures for the approval of materials and products for water supply systems [119,120]. In 2012 Belgium also issued independently a document for acceptance of materials in contact with drinking water [121], while some countries like Portugal and Slovenia mainly follow the requirements set by 4MS [120]. They include lists of allowed composition for cement and its additives, organic materials (e.g., polyethylene (PE) and its derivates—PEX, GFRP, and rubber) and metals (e.g., copper and its alloys; Cu-Zn, Cu-Zn-As, Cu-Zn-Pb, Cu-Zn-Pb-As, etc.). The document recommends also standard procedures for testing the materials adequacy in contact with water, to avoid possible corrosion and microbial growth promotion. Materials more prone to corrosion negatively affect residual chlorination and can be thus used only for water with pH ≥ 7.5, concentration of Ca^2+^ ≥ 0.5 mmol/L and free CO_2_ ≤ 0.25 mmol/L, and conductivity ≤600 µS/cm (measured at 25 °C) [119,120]. Materials should not promote the growth of planktonic cells of total coliforms at 37 °C and total microbial count at 22 °C and the establishment of biofilms should be limited under test conditions [120]. Studies conducted in the last decades have shown a certain correlation between used materials and the establishment of biofilms [2]. Although biofilms occur independently of the hydrophobicity or hydrophilicity of the material [122], it was noted that both bacteria and fungi were more likely present in pipe systems made of steel or iron, in comparison to PVC [28,123,124,125]. One of the reasons has been the chemical interaction between metals and free-chlorine leading to corrosion and the loss of residual effect of free-chlorine [2,28]. Subsequently, surfaces of such materials become rough, inducing changes in water flow and causing the reduction in shear forces, enabling easy attachment of microorganisms [126].

Microbial biofilms are formed in 3 stages, starting with initial colonizers irreversibly attaching on inorganic and organic surface molecules. In the second stage, secondary microbial colonizers attach to the initial colonizers and synergistically form the mature biofilm [127]. Only ~15% of a biofilm is represented by microorganisms, while the rest of the biofilm is composed of extracellular polysaccharides (EPS), water, proteins, nucleic acids and lipids [124]. During the last stage of the maturation process, microorganisms from the upper part of biofilm are released into water [128]. While initial colonizers are mainly bacterial species, secondary colonizers also include protozoa and fungi. The role of fungi in biofilms is still poorly investigated; however, it was suggested that they may provide bacteria with intermediate decomposition products that they cannot produce on their own [129]. Fungi are also involved in building up the extracellular polymeric substances of a biofilm, such as humic acids and aliphatic constituents (carbohydrates and peptides) [130]. Fungal hyphae and pseudo-hyphae, formed during the biofilm maturation, cross-link the biofilm structure, making the latter more difficult to remove and present a scaffold for the attachment of bacteria [124,131]. The number of fungal cells inside biofilms may be up to 5000 times higher than in running water, with filamentous fungi being more likely present than yeasts [28]. Experimentally, the formation of fungal biofilms was studied with single strains for the yeast genera *Candida*, *Saccharomyces*, *Naganishia* (former *Cryptococcus*) and *Aureobasidium*, and filamentous fungal genera *Aspergillus*, *Penicillium*, *Coriolus* and *Trichoderma*; many of which are frequently present in drinking water (Table 1) [21,31,131]. Fungal biofilms were fully formed within 48 h from the beginning of an experiment mimicking real conditions in tap systems [132]. The presence of fungi in in vivo biofilms from tap systems in private homes, hospitals or industrial network was confirmed for opportunistic and pathogenic species from the genera *Aspergillus*, *Candida*, *Exophiala*, *Fusarium*, *Malassezia*, *Ochroconis*, *Penicillium*, *Phialophora*, *Phoma* and *Rhinocladiella* [23,26,27,31,133,134]. Once established, biofilms are difficult to be fully removed from the pipe system, which on the long-term leads to altered taste and odour of water, production of allergenic or irritating compounds, and mycotoxins with an effect on human health (Figure 1) [2,21]. 

### 2.8. Commonly Used Methods for Isolation and Detection of Fungi in Water and Biofilms

Results for fungi obtained from water habitats may vary among different studies; reason being the lack of a uniform approach for detection or isolation of fungi. Isolation methods for fungi from water are generally based on water filtration followed by either conventional microbiology cultures or molecular approaches [21]. 

The first step includes sampling of water in sterile plastic or glass containers, with different studies using different volumes of water for filtration. In our review of published reports, volumes for sampling drinking water ranged from 50 mL to up to 1 L [8,13,19,135,136]. Filtration was usually performed with the use of sterile cellulose filters, with porosity between 0.2 µm and 0.45 µm; 0.45 µm diameter being recognised as the most efficient one [21,133]. Filters were then placed onto solid agar media, frequently supplemented with an antibiotic to prevent the bacterial growth. Since the choice of media is not defined, they may vary from oligotrophic to nutrient-rich; some authors used also selective media supporting the growth of targeted fungal genera. Most commonly reported media were Sabouraud dextrose agar (SDA), Sabouraud glucose agar (SGA), Sabouraud gentamicin-chloramphenicol agar (SGCA), malt extract agar (MEA), corn meal agar (half-strength) (CMA/2), Czapek Dox agar (CZ), potato dextrose agar (PDA), Dichloran Rose Bengal chloramphenicol agar (DRBC), Neopeptone glucose Rose Bengal aureomycin agar (NGRBA), Dichloran 18% glycerol agar (DG18), erythritol-chloramphenicol agar (ECA), tap water agar and oomycete selective medium [8,13,19,21,26,133,134]. Most of these support growth of filamentous fungi, whereas DRBC, DG18 and ECA were used to obtain yeasts and black yeasts from both, water and biofilm samples [19,21,26,64,133]. Incubation was also reported at different temperatures (20, 25, 30 or 37 °C), for 3 days to up to 4 weeks. The broadest spectrum of fungi was reported at 30 °C after 14 days [21]. Pure fungal cultures were obtained and identified per macro- and micromorphological features. Some studies conducted during the last decade also used molecular approaches (polymerase-chain reaction and sequencing). The generally recommended genetic marker for basic fungal identification is the whole internal transcribed spacer (ITS) region (the official fungal DNA barcode) [137,138], which has already been used in most studies [2,19,26,67,134]. Considering the limitations of the ITS in separating all fungal species, when used on its own as primary fungal DNA barcoding region, more recently the elongation factor 1 alpha has been added as secondary [139].

Sampling of biofilms has usually been performed with scraping or swabbing surfaces; with a generally recommended surface area of 1 cm^2^ [21,26,27,67]. Obtained biofilm material was then either plated onto solid media directly from a swab, or firstly resuspended in sterile buffer or saline solution, followed by 100 µL of the suspension being plated onto the medium using the spread plate technique [21,26,27,67]. Some authors successfully obtained fungi after putting pieces of pipe material together with the biofilm directly onto media. However, the disadvantage of the method is its difficulty in repeating the experiment, since that part of the pipe isreplaced after sampling [133]. For this reason, Siqueira et al. [133] recommended the use of “sampler devices” instead—PVC pipes within polyethylene or acetate coupons that can be placed in the pipe network allowing biofilms to grow inside the device, without removing the original pipe [133]. Media used, incubation conditions and identification of pure fungal cultures from biofilms were usually the same as described above for planktonic fungi in water samples [19,21,26,67,133].

Culture-dependent methods may give a general overview over the presence of cultivable fungi from water and biofilms. However, results vary significantly and are usually limited by the choice of growth media, temperature and incubation time [21,133]. Culture-independent methods have thus gained relevance, either as a support to the classical methods, or to detect and quantify fungal DNA directly in water; e.g., Real Time Quantitative PCR [140,141]. Few studies used a metagenomic pyrosequencing approach for the detection of fungi in tap water or biofilm samples [19,23,27,67]. Since all of them used different kits for DNA extraction, different oligonucleotide pairs and different sequencing techniques (TEFAP, 454 Platform), their results are hard to compare. However, authors reported differences in the results obtained via metagenomic analyses in comparison to culture-based techniques. Metagenomic approaches usually yield higher fungal diversity, but also reveal different percentages of single species in biofilms [19,23,27,67]. Further investigation on metagenomic approaches should be conducted to select the best fungal detection in water and biofilm; including optimization of environmental DNA extraction, choice of primers and sequencing techniques used (e.g., TEFAP, 454 Platform, Illumina, Ion Torrent, etc.)

## 3. Exposure to Fungi from Water in Indoor Environments and Their Medical Relevance

Although the number of fungal cells may significantly vary, and is not necessarily high in running drinking water, water is still a vector for fungal particles to reach human-made indoor habitats; where fungi are exposed to environmental pressure, leading towards the selection of opportunistic human pathogens [19,21]. People may come across them on a daily basis at different exposure points; directly while using water for drinking, bathing and showering, or indirectly due to the use of appliances connected to the water supply, for instance dishwashers and washing machines (Figure 1) [19,27,67,80].

Over the last two decades, the increasing number of immunocompromised patients led to an increase in the incidence of nosocomial and community-acquired infections by opportunistic fungal pathogens. Fungi can enter the hospital environment and may survive and proliferate, especially in humid and unsterile areas. Of special concern is direct or indirect exposure of immunocompromised individuals to water-borne fungi from the environment, to single fungal propagules, as well as to fungi in biofilms potentially formed in catheters, dental units, haemodialysis units and intensive care units [21,31,136,142,143]. Severe invasive fungal infections have a high mortality rate, currently estimated at between 50% and 100%; depending on the species involved [2,144].

Table 2 intends to summarize the most common fungal genera/species isolated from different water sources in Europe, recognised as causative agents of opportunistic infections and their effect on human health. The following paragraphs describe some of these fungal genera, their occurrence in water supplies and possible health effects.

### 3.1. Direct Contact with Fungi

People come in direct contact with fungi from water via skin and mucosa when bathing and showering. Indoor surfaces in regular contact with tap water (e.g., bathrooms) are colonised mainly with opportunistic pathogens. Among these the most frequently isolated filamentous fungi belong to the genera *Cladosporium*, *Fusarium*, *Ochroconis*, *Phoma* and *Scedosporium*, yeasts of the genera *Candida*, *Cryptococcus* and *Rhodotorula*, and black yeast from the genera *Aureobasidium*, *Cladophialophora*, *Exophiala* and *Rhinocladiella* [181,182,183,184]. The origin of their spores could be the tap water but they are also common in the air. After deposited, spores start to germinate. Spores of species adapted to high water activity can colonize surfaces covered by water (bathroom surfaces, sink, etc.), while those adapted to low water activity thrive on hydrophilic surfaces (i.e., in between ceramic tiles). Organic materials found in bathrooms and kitchens (dust, building materials) serve as nutrient supply—some of those fungi can degrade and utilize detergents and soaps [185].

Recent research conducted on shower hose biofilms revealed the presence of the following opportunistic pathogens: *Aspergillus glaucus*, *Cladosporium* spp., *Exophiala mesophila*, *Fusarium fujikuroi* species complex, *Malassezia restricta*, *Penicillium* spp. and *Schizophyllum commune* [27]. During showering people are exposed to fungal propagules also via watery aerosols released into the environment (Figure 1) [21]. Their inhalation is the most relevant route of systemic infection for susceptible patients. Any situation that enhances the air-borne dispersion of mould propagules increases the exposure of patients to such pathogens [142]. Thus, special attention should be paid to aerosols released in bathrooms in hospital environments. Anaissie et al. [181] reported a change in the microbial community in the air and on surfaces between and immediately after showering. Showering increased the presence of filamentous fungi from the genera *Alternaria*, *Acremonium*, *Aspergillus*, *Cladosporium*, *Fusarium*, *Paecilomyces*, and *Penicillium*, regularly involved in worsening of asthma symptoms, hypersensitivity pneumonitis and skin irritation [31,181]. Molds were recovered in 70% of 398 water samples. The authors found that hospital water distribution systems may serve as a potential indoor reservoir of *Aspergillus* and other molds, leading to aerosolization of fungal spores and potential exposure for patients. In a study performed by Warris et al. [186], water was identified as the source of exposure in a nosocomial outbreak. In fact, the genotype of *A. fumigatus* recovered from water was related to the genotype of isolates collected from three patients. Environmental *A. fumigatus* isolates resistant to azoles have been described in recent years especially in Europe [187]. The exposure of immunocompromised patients or persons with a hyper-reactive immune system to these resistant strains may lead to serious invasive fungal infections, difficult to manage due to the lack of response to the available antifungals. Patients inhale both susceptible and resistant conidia, but the resistant conidia may have a selective advantage, thus allowing their germination in the lungs and subsequently causing an invasive disease. 

Some fungi like *Fusarium* are particularly adapted to an aquatic environment and are present in water worldwide as part of biofilms. *Fusarium* species cause a broad spectrum of infections in humans, including superficial and locally invasive diseases. The principal portal of entry for *Fusarium* spp. are the airways, followed by the skin at the site of tissue breakdown and possibly the mucosal membranes [188]. The clinical form of fusariosis depends largely on the immune status of the host and the portal of entry, with superficial and localized disease occurring mostly in immunocompetent patients and invasive and disseminated disease affecting immunocompromised patients. Further, and on a global scale, *Fusarium* is also one of the most common etiological agents of fungal corneal ulcers [189,190,191]. 

*Like Fusarium*, *Scedosporium* spp., especially *S. apiospermum*, *S. aurantiacum* and *L. prolificans* (former *S. prolificans*), are also saprophytic fungi isolated worldwide from soil, plant residues and polluted waters. These species usually cause localized disease after penetrating trauma or aspiration of polluted water. However, in immunocompromised patients they may cause severe pulmonary or disseminated infections. Recently, *S. apiospermum* has been isolated from patients with chronic lung disease, receiving chronic corticosteroid therapy, in particular in cystic fibrosis patients [192].

### 3.2. Indirect Contact with Fungi

Indirectly, people are exposed to fungi from water via everyday use of home appliances, using water for their operation (Figure 1). Examples of such are dishwashers and washing machines, where fungi from water are exposed to extreme life conditions like elevated temperatures, use of detergents and drastic pH changes [58,80]. Environmental pressure inside the appliances leads to the selection of polyextremotolerant water-related fungi, with many of them being recognised as opportunistic pathogens [58]. Recent discoveries of fungal colonization of domestic dishwashers showed great consistence in fungal biota. Globally, dishwasher rubber seals were colonized with muriform black yeasts *Exophiala dermatitidis* and *E. phaeomuriformis*, *Candida parapsilosis*, *Rhodotorula mucilaginosa*, and filamentous *Magnusiomyces capitatus*, *Fusarium dimerum*, *F. oxysporum* and the *F. solani* species complexes [58,67]. Except *M. capitatus* the above listed fungi colonizing dishwashers originated from water sources. While tap water contained between 1–130 fungal CFU/L, the number inside dishwasher biofilms increased to 10^2^–10^6^ CFU/cm^2^ [19,67]. Enrichment of water-related fungi inside dishwashers may represent a risk for human health due to the use of contaminated dishes and via aerosols released after completed washing cycles. As proven, dishes were rarely colonised with fungi, but aerosols released from dishwashers contained fungi of the core mycobiota—*C. parapsilosis*, *R. mucilaginosa* and *E. dermatitidis*, as well as water- and air-related filamentous fungi from the genera *Aspergillus*, *Cladosporium*, *Penicillium* and *Trichoderma* [67]. Aerosols from dishwashers contributed to contamination of kitchen surfaces when kitchens with dishwasher were compared to kitchens without them [67]. 

Similar to dishwashers, selection of certain water-related fungi happens also in washing machines. Recent ecological trends support washing at lower temperatures, 40 °C being the choice of most consumers [80]. Besides, use of biodegradable detergents and softeners leads to the formation of slimy film on plastic and rubber parts of washing machines, offering an ideal environment for biofilms [80,193]. Water-related fungi representing the core mycobiota of washing machines differed from those colonising dishwashers. Washing machine mycobiota consisted primarily of *F. oxysporum* species complex, followed by *C. parapsilosis*, *R. mucilaginosa* and black yeast *E. phaeomuriformis* [80,194]. In comparison to dishwashers, washing machines favoured colonisation of mesophilic water-related fungi *E. mesophila*, *E. lecanii-corni*, *Ochroconis* spp. and *Penicillium* spp., together with previously reported *Mucor* spp. and *Trichophyton mentagrophytes* [80,193]. Besides causing odour in washing machines and clothing, enrichment of water borne fungi may pose a health risk due to the contact of contaminated clothes with skin [193].

Members of the genus *Exophiala* are dematiaceous fungi widely distributed in the environment, especially in the soil, wood, polluted water, and sewage. Humid indoor environments lead to the selection of only few mesophilic and thermotolerant opportunistic species, such as *E. dermatitidis*, *E. phaeomuriformis*, *E. mesophila*, and *E. lecanii-corni* [67,80]. Besides dishwashers and washing machines, also steam baths provide optimal growth conditions for *E. dermatitidis* and *E. phaeomuriformis* [195]. *Exophiala* can cause post-traumatic cutaneous infections, keratitis, onychomycosis, otitis externa, it can infect lungs of patients with cystic fibrosis, and cause disseminated mycosis in immunocompromised patients, even involving the brain [32]. 

*Candida* was the second most common fungal genus, isolated from the above mentioned indoor habitats. *C. albicans* and *C. parapsilosis* currently show up in the first ranks of the list of potential hospitalization threats on a worldwide scale [196,197]. Both are associated with biofilm formation and are commonly found in water collected from hospitals and private homes [19,67,80], indicating that water may be one of the means of propagation and a possible cause of nosocomial infections. 

### 3.3. Fungal Metabolites—Mycotoxins, Allergens, Microbial Volatile Organic Compounds (MVOCs)

Not only fungi can cause adverse health effects, but also their secondary products are involved in those effects. Exposures include also those to allergens, airborne cell wall components and metabolites such as MVOCs, and mycotoxins (Figure 1). Many metabolites are candidates for causal agents that exhibit allergenic, cytotoxic, irritant, immuno-modulatory and psychosomatic effects [198,199,200]. A significant number of allergenic fungi have been reported from water (Table 2), but to our knowledge, there are no reports on allergic symptoms caused by fungi in tap water. Exposure of humans or animals to mycotoxins can cause severe health problems. Some mycotoxins are considered to be carcinogenic [201]. They have been shown to exacerbate airway hyper-reactivity, inflammation, and remodelling by both ingestion, and inhalation in a murine asthma model [30,202]. However, recent findings implicate that increased exposure to secondary fungal metabolites does not explain the elevated risk of asthma development in homes in association with moisture damage [203].

Exposure to mycotoxins is likely to occur from food, water or beverages made with water. Mycotoxins may be aerosolized and further inhaled [30,202]; if present in water and as proved in several occupational environments [204,205,206,207,208]. In addition, Boonen et al. [209] reported that aflatoxin B1 can penetrate into and through skin, thus the contact with liquids containing this mycotoxin should be avoided [209]. The estimated values of secondary fungal metabolites through ingestion are considerably higher than by inhalation, but compared to the exposure to secondary metabolites through foods, these total amounts are marginal [203]. Kelley et al. [104] showed that mycotoxins can be produced during submerged growth in water, but normally the levels of mycotoxins would be low. There is a lack of information about the effect on health of fungi being ingested directly with drinking water from the tap [21]. However, possible threats may be presented by taps that supply water not used on a daily basis; or contaminated bottled water stored for longer time in plastic bottles (Figure 1) [66,87]. A few studies conducted in Europe on bottled water reported the presence of fungi, with the genera *Aspergillus*, *Aureobasidium*, *Cladosporium*, *Debaryomyces*, *Exophiala*, *Fusarium*, *Paecilomyces*, *Penicillium*, *Talaromyces*, and *Trichoderma* being the most commonly detected (Table 1). These genera are known to form biofilms on plastic and can use plastic material as the sole source of carbon [182]. Their growth inside bottled water may lead to mycotoxin production affecting human health (Table 2) [87]. Therefore, the existence of fungal species in drinking water that potentially can produce mycotoxins is an issue of concern and needs further studies [203]. 

## 4. Discussion

Drinking water in European countries originates either from surface water or groundwater [2,4,42]. At the beginning of 19th century drinking water in urban areas was available with little or no purification needed, but growing industrialization and urbanization led to increased pollution and occurrence of faecal-borne diseases [42]. Recent knowledge of ecology and transmission routes of faecal microorganisms promoted the development of water cleaning processes, such as filtration and chlorination [42]. The process of water cleaning evolved throughout time, including new techniques such as aeration and ultra-filtration [22]; chlorine remains the most used agent for chemical disinfection providing also the residual effect [42].

Based on past knowledge, countries worldwide still use faecal-borne microorganisms as indicators for water pollution [37], but considering the hygiene standards and conditions in developed countries changed considerably along time, quality assessment parameters for drinking water safety should be updated to reflect the present situation. While during the 19th and beginning of the 20th century water consumption was low and more or less limited to drinking and food preparation [42], it is today used in larger volumes also for daily hygiene, including showering, dishwashing and laundry [27,67,80]. Urbanisation, dense population in cities and especially the development of new daily routines (also the use of new, human-made materials, such as plastic, rubber, and metal coats) [58,71]. In parallel with higher hygiene standards and ecological concerns, the use of low water temperatures and biodegradable cleaning agents created specific niches which select and support the enrichment of stress tolerant microbial species, able to form biofilms and degrade new materials [58,67,80]. Among them, fungi showed remarkable adaptability to changes in living conditions and are becoming regularly detected in the metropolitan environments associated to higher density populations, man-made materials and complex chemical compounds [58,67,71,80].

Due to high adaptability at a physiological level, fungi may colonise environments with extreme growth conditions, one of them being also oligotrophic water systems [2]. Presence of fungi in natural raw water sources was investigated mainly in the relation with plant diseases and microbial blooms [44,60]; and connected to diverse conditions supporting their growth, such as presence of certain ions, changes of pH, temperature, sunlight and organic material [2,4,12,19,43,45]. 

Despite well-developed raw water cleaning processes, fungi were discovered in tap water systems in single-cell form and as a part of biofilms [2]. During the last 30 years, researchers from 19 European countries investigated and reported the presence of fungi in a relation to surface water, groundwater and tap drinking water (Table 1). A variety of fungal genera, with more than 400 different species, was found to inhabit different water sources. The most commonly detected fungi belonged to the genus *Aspergillus*, reported from 17 out of 19 countries (89.5%), followed by *Cladosporium* and *Penicillium* species (both were reported from 84.2% of countries), *Trichoderma* (73.7%), *Alternaria* and *Fusarium* (both 68.4%) and *Aureobasidium* and *Mucor* (both 52.6%) (Table 1). The majority of the listed genera were isolated from both raw water sources (surface- and groundwater) and tap water, while species from the genera *Mucor*, *Trichoderma*, and *Penicillium* were more related to surface water samples (Table 1). This research was conducted mainly using traditional cultivation techniques and may thus not be exhaustive [21]. 

Culture-based methods are often biased by the selection of culture media [210]. Moreover, dead microorganisms are not culturable even though they may retain activity linked to allergenic proteins or toxic secondary metabolites [211]. On the contrary, DNA-based techniques can detect also unculturable, dead and dormant microorganisms. Polymerase chain reaction (PCR) amplifies DNA markers of interest and is highly sensitive to detect down to one fungal spore from an environmental sample [212]. In the last decade, also high-throughput sequencing (HTS) methods have been introduced to analyse fungal communities in the environments [213]. These are not quantitative, but can be combined with quantitative PCR (qPCR) to provide taxon-specific concentrations of fungi [214], and thus be used for taxon-specific measurements of water-borne fungi. This is of crucial importance for fast detection of species of interest, particularly in hospital environment, where the above listed fungal genera are not only the most frequently reported in drinking water, but are also often being recognised as causative agents of diseases (Table 2) [215,216,217,218]. 

Since the European population is becoming on average older and the ratio of immuno-compromised people is increasing, also fungal infections are becoming regularly present, not only in hospitals, but also in private homes [219]. Human immune impairment may be transient (acute) or permanent (chronic), and is not always deriving from immune-suppression. Hyper-reactivity of the immune system also potentiates fungal colonization and pathogenesis [220,221]. Under this category fall the people who suffer from chronic bronchitis and asthmatic disorders [222]. Some conditions may even be triggered or sustained by fungal colonisation (i.e., allergic bronchopulmonary mycosis), be it caused by the usually overlooked *Candida* spp. [223], *Aspergillus* spp. or by quite a few other fungal agents (Table 2) [219]. Populations prone to fungal infections, include also individuals with transient conditions or situations (e.g., pregnancy), chronic illnesses, such as diabetes mellitus, or circulatory system impairments (which mitigate a good blood circulation in lower body extremities and peripheral tissues e.g., skin and toe or fingernails). The latter group also includes individuals suffering from chronic pulmonary obstructive disease (COPD), cystic fibrosis, uncontrolled (un-medicated) HIV, cancer and those who use immunosuppressive drugs and therapies [219,224]. All these individuals do not inhabit hospitals only, but are in fact more likely present in their private homes due to patient and bed management policies and costs, and most definitely to avoid exposure to nosocomial infection agents and multi-drug resistant microbes [224]. Should then fungal contaminants in drinking water supply be of concern as a general concept? How cost effective would this activity be?

Drinking water quality management is shifting towards a risk-based approach worldwide. The 4th Edition of the WHO Guidelines on Drinking Water Quality [34] considers end-point testing in itself “too little-too late” as it only gives information on the quality of water, which was already consumed, and only focuses on known or regulated contaminants. Therefore, relying solely on monitoring provides limited protection for human health. The water safety plan approach, on the other hand, calls for the identification of all hazards throughout the water supply system and the management of associated risks before they reach the consumers. Fungi, as previously unrecognized risk factors, fit very well in this concept, and should be considered in water safety planning on both the water supply and the building water system level; especially in high-risk settings. Guidelines exist in many European countries to develop water safety plan for health-care facilities as a tool in the prevention of nosocomial infections [225]. Hazard identification should extend to fungi by considering how can they enter to and colonize the water system. For raw water derived fungi, the efficiency of treatment technologies in their removal is the key issue, as described above. Certain technological steps, such as aeration, may also contribute to the fungal load. Regrowth of fungi may occur in the water distribution system, especially in premise plumbing, where the above listed factors favouring biofilm formation, such as ambient temperature and low flow, are most likely to be present. Risk management interventions, which were demonstrated to be efficient against other pathogens residing in water system biofilms, such as *Legionella*, may also provide some protection against fungi, but further data is necessary to support this assumption.

## 5. Conclusions

Recent discoveries on fungi requiring special attention include the presence of opportunistic and emerging pathogens in raw water sources. Many environmental species (particularly of the genus *Aspergillus*) recently display resistance to azoles, being the target of many studies as a serious health risk. In addition, many water-borne fungi showed resistance to the usual water disinfection procedures, allowing them to enter water distribution systems; where they form mixed biofilm communities with bacteria, algae and protozoa. Biofilms increase ability to survive heat- and chlorination-shocks. Consequently, fungal presence in tap water distribution systems leads to the enrichment of the sturdiest fungi tolerating 37 °C, in certain water-related indoor environments (e.g., dishwashers, washing machines, bathrooms and showers). Enrichment of fungi in indoor environments may affect human health via direct exposure, such as inhaling of aerosols, contact or through drinking; and indirectly by exposure to contaminated surfaces, dishes or clothes. Thus, the present knowledge of ecology and pathogenesis of fungal contaminants in water reveals the need to measure and regulate their presence in drinking water at least in the environment with high numbers of immunocompromised people.

The authors of this white paper conclude that the herein gathered reports of fungal contaminants in drinking water, as many other possible inlays and invasive activities, illustrate and justify a recommendation to consider fungi in risk assessment and risk management of drinking water, including monitoring in relevant settings.

### 5.1. Future Scientific Research Needs

During the production of this white paper, knowledge gaps were identified on the following items:

1. Development of a consensus standard operating analytical procedure for the assessment of fungal contaminants in drinking water;
2. Establishment of a geographically broad report on fungal contaminants in water (enumeration and variety) using a standardized analytical procedure.
3. Development of sampling techniques necessary to detect sporadic particles released by biofilms.
4. Large scale assessment of the presence and quantification of mycotoxins and MVOCs in drinking water.
5. Generating agent specific epidemiological assessments of the health effects resulting from drinking-waterborne fungi.

### 5.2. Recommendations

1 Surveillance of drinking water in relevant contexts.
2 Adoption of the current Swedish legislation with an update of its fungal parameters to levels compatible with current knowledge.
3 Special attention to be paid to hospitals and other open-to-public buildings, where immunocompromised people circulate or stay for a longer time and where molecular typing may be required in order to track sources or link infections together.

### 5.3. Afterword

The Swedish drinking water regulation [226] determines:

- Filtration: use of filters with a pore diameter of 0.45 μm and a filtration volume of 100 mL
- Media: Rose Bengal Chloramphenicol and Chlortetracycline Agar (RBCC) for filamentous fungi and for yeasts
- Incubation temperature: 25 °C.
- Incubation time: 7 days
- Results: maximum allowed number of moulds + yeasts = 100 CFU/100 mL [41]

The consensus modified version and justification:

- Filtration: use of filters with a pore diameter of 0.45 μm and a filtration volume 100 mL
- Media: Sabouraud agar for filamentous fungi and Dichloran Rose Bengal Chloramphenicol Agar (DRBC) for yeasts
- Incubation temperature: 30 °C yields the highest diversity as reported by different authors
- Incubation time: 7 days
- Results: maximum allowed number (Unchanged due to the lack of epidemiological data that could support alterations) of moulds + yeasts = 100 CFU/100 mL
- Detection and quantification of clinically relevant species/genera (culture-based + PCR-based in hospitals and other open-to-public buildings)

Quantitative analysis of the fungal agents listed in Table 2 would be the ideal solution, but ultimately, rather labour-intensive and costly. It is, however, not unprecedented: In 1996, a recommendation from the American Industrial Hygiene Association states that “the presence of the species *Stachybotrys chartarum*, *Aspergillus versicolor*, *Aspergillus flavus*, *Aspergillus fumigatus* and *Fusarium moniliforme* in different settings requires the implementation of corrective measures” [227]. 

Certain areas of hospitals, for which a strict surveillance is recommended, are units where the most susceptible patients are temporary residents: Intensive care units (due to open wounds and burns), infectious diseases wards, haematology, oncology and transplant units. Patients must not be exposed to fungal contaminants in drinking water in these units. Molecular methods may be considered for species identification, but they carry the usual issue of looking into genetic material instead of at viable organisms. When combined with classical identification methods, they can support source tracking of any relevant colonies by typing. This is of great importance in a hospital in order to promote the mitigation of nosocomial infections. Therefore, as a future research, authors emphasize the necessity of the development of DNA-based, routine test(s) for waterborne fungi.

## Figures and Tables

**Table 1:** Fungal genera and species isolated from groundwater, surface water, tap water and non-mineral bottled water reported in studies conducted in Europe during the last 30 years.

Fungal Species	BSL *	Water Type				Country	Reference
		Ground Water	Surface Water	Tap Water	Non-Mineral Bottled Water		
**Ascomycota (phylum)**							
*Acremonium psammosporum*	1	+	−	+	−	Germany	[11]
*Acremonium* spp.	1/2	+	+	+	−	Germany, Greece, Slovakia, France, Austria, Portugal, Norway, Belgium, Serbia, UK, Sweden, Hungary	[5,6,7,8,9,10,11,12,13,14,16,18,79]
*Acrostalagmus luteoalbus*	1	+	+	+	−	Germany, Serbia	[11,12]
*Alternaria alternata*	1	+	+	+	−	Austria, Portugal, Ukraine, Serbia, Slovenia, UK, Hungary	[9,12,14,15,17,79,80]
*Alternaria atra*	1	−	+	−	−	UK	[9]
*Alternaria botrytis*	1	−	−	+	−	UK	[9]
*Alternaria infectoria*	1	−	+	−	−	Portugal, UK	[9,15]
*Alternaria* spp.	1	+	−	+	−	Greece, Slovakia, Portugal, Norway, Hungary, Belgium, Spain, Germany, UK	[7,8,9,10,13,16,18,23,81,82]
*Alternaria tenuissima*	1	−	−	+	−	Hungary	[79]
*Arthrinium phaeospermum*	1	−	+	+	−	Norway, UK	[9,20]
*Arthrobotrys* spp.	1	+	−	−	−	Slovakia	[7]
*Arthrographis* spp.	1/2	−	−	+	−	Poland, Norway, UK	[9,10,66]
*Ascochyta* spp.	1	−	−	+	−	UK	[9]
*Aspergillus aculeatus*	1	−	+	−	−	UK	[9]
*Aspergillus alliaceus*	1	−	+	−	−	Portugal	[15]
*Aspergillus brasiliensis*	1	−	+	−	−	Portugal	[15]
*Aspergillus calidoustus*	1	−	+	+	−	Portugal, Norway	[18,20]
*Aspergillus candidus*	1	−	+	−	−	Serbia	[12]
*Aspergillus carbonarius*	1	−	−	+	−	Greece	[8]
*Aspergillus chevalieri*	1	−	+	−	−	Portugal	[15]
*Aspergillus clavatus*	1	+	+	+	−	Norway, UK	[9,20]
*Aspergillus fischeri*	1	−	−	+	−	Slovenia	[80]
*Aspergillus flavus*	2	+	+	+	−	Germany, Greece, Belgium, Serbia, UK	[8,9,11,12,16]
*Aspergillus fumigatus*	2	+	+	+	+	Germany, Greece, Poland, Hungary, Norway, Portugal, The Netherlands, Finland, Belgium, Serbia, UK	[8,9,10,11,12,15,16,18,20,28,66,83,84,85]
*Aspergillus glaucus*	1	−	−	+	−	Greece	[8]
*Aspergillus inflatus*	1	−	+	−	−	Norway	[20]
*Aspergillus insuetus*	1	+	−	−	−	Portugal	[18]
*Aspergillus japonicus*	1	−	+	−	−	UK	[9]
*Aspergillus nidulans*	1	−	−	+	−	Greece, Belgium	[8,16]
*Aspergillus niger*	1	+	+	+	−	Germany, Greece, Poland, Norway, Belgium, Ukraine, Serbia, UK, Portugal	[8,9,10,11,12,16,17,18,20,28]
*Aspergillus ochraceus*	1	−	−	+	−	Greece	[8]
*Aspergillus ostianus*	1	−	−	+	−	Greece	[8]
*Aspergillus parasiticus*	1	−	−	+	−	Greece, Poland	[8,28]
*Aspergillus parvulus*	1	−	−	+	−	UK	[9]
*Aspergillus repens*	1	+	−	−	−	Portugal	[18]
*Aspergillus restrictus*	1	+	−	+	−	Greece, The Netherlands	[8,85]
*Aspergillus sydowii*	1	−	+	+	−	Norway, Belgium	[16,20]
*Aspergillus terreus*	1	+	+	+	−	Greece, Austria, Portugal, Norway, UK	[8,9,10,14,15,18]
*Aspergillus tubingensis*	1	−	+	−	−	Portugal	[15]
*Aspergillus ustus*	1	+	+	+	−	Poland, Norway, Portugal, Serbia	[12,15,20,28]
*Aspergillus versicolor*	1	+	+	+	+	Germany, Poland, Serbia, Slovenia, UK	[9,11,12,28,80]
*Aspergillus viridinutans*	1	−	+	−	−	Portugal	[18]
*Aspergillus* spp.	1/2	+	−	+	−	Slovakia, France, Austria, Portugal, Norway, Spain, Slovenia, Hungary	[5,7,10,13,14,19,79,81]
*Asteroma* sp.	1	−	+	−	−	UK	[9]
*Asteromella* sp.	1	−	−	+	−	UK	[9]
*Aureobasidium melanogenum*	1	+	−	+	−	Slovenia	[19,67,80]
*Aureobasidium pullulans*	1	+	+	+	+	Greece, Norway, Austria, Ukraine, Serbia	[8,12,14,17,20,86]
*Aureobasidium* spp.	1	+	+	+	−	Slovakia, UK, Portugal, Hungary	[7,9,18,79]
*Beauveria bassiana*	1	+	+	+	−	Norway, Austria, UK, Portugal	[9,14,18,20]
*Beauveria brongniartii*	1	−	+	−	−	Norway, UK	[9,20]
*Beauveria* spp.	1	+	−	−	−	Slovakia	[7]
*Bionectria ochroleuca*	1	+	−	−	−	Portugal	[18]
*Bionectria* sp.	No data	+	−	−	−	Portugal	[18]
*Bipolaris* spp.	1/2	−	−	+	−	Greece	[8]
*Biscogniauxia* sp.	No data	−	+	−	−	Portugal	[18]
*Bisifusarium dimerum*	1	+	−	+	−	Norway, Slovenia	[19,20,67]
*Boeremia exigua*	1	−	−	+	−	UK	[9]
*Botryotrichum* spp.	1	+	−	−	−	Slovakia	[7]
*Botrytis cinerea*	1	−	+	+	−	Norway, Portugal, Serbia, UK	[9,12,15,20]
*Botrytis elliptica*	1	−	+	−	−	Norway	[20]
*Byssochlamys lagunculariae*	1	−	+	−	−	Norway	[20]
*Cadophora luteo-olivacea*	1	+	−	−	−	Germany	[23]
*Cadophora malorum*	1	+	+	+	−	Germany, Poland, Norway, Austria	[14,20,23,28]
*Cadophora melinii*	1	−	+	−	−	Norway	[20]
*Candida albicans*	2	−	+	−	−	Ukraine	[17]
*Candida glaebosa*	1	−	−	+	−	Slovenia	[67]
*Candida intermedia*	1	−	−	+	−	Poland, Slovenia	[66,67]
*Candida orthopsilosis*	2	−	−	+	−	Slovenia	[19]
*Candida parapsilosis*	2	+	−	+	−	Poland, Slovenia	[19,66,67]
*Candida pararugosa*	1	−	−	+	−	Slovenia	[19,67]
*Candida pseudointermedia*	1	−	−	+	−	Slovenia	[19]
*Candida saitoana*	1	−	−	+	−	Slovenia	[19]
*Candida sake*	1	−	+	−	−	Portugal	[15]
*Candida* sp.	No data	+	−	+	−	Portugal, Greece	[8,15]
*Candida tropicalis*	2	−	−	+	−	Greece	[8]
*Candida versatilis*	1	−	−	+	−	Poland	[66]
*Capronia munkii*	1	−	+	−	−	Portugal	[18]
*Capronia pilosella*	1	−	−	+	−	Germany	[23]
*Capronia* sp.	No data	−	−	+	−	Slovenia	[67]
*Cephalosporium* spp.	1/2	+	+	+	−	Slovakia, Portugal	[7,18]
*Ceratocystis fimbriata*	1	−	+	−	−	Norway	[20]
*Chaetomium globosum*	1	−	+	−	−	Norway, Serbia, UK	[9,12,20]
*Chaetomium* spp.	1	+	−	+	−	Greece, Norway, Portugal	[8,13,20]
*Chalara* sp.	No data	+	−	+	−	Germany	[11]
*Chalaropsis* spp.	1	+	−	−	−	Slovakia	[7]
*Chrysosporium* spp.	1	−	−	+	−	Greece	[8]
*Chrysonilia* sp.	No data	+	+	−	−	Norway	[20]
*Cistella acuum*	1	+	−	+	−	Austria	[14]
*Cladosporium cladosporioides*	1	+	+	+	−	Germany, Greece, Poland, Norway, Portugal, The Netherlands, Serbia, Slovenia, UK, Hungary	[8,9,11,12,15,18,20,23,28,79,80,85]
*Cladosporium cucumerinum*	1	−	+	−	−	Serbia	[12]
*Cladosporium diaphanum*	1	−	+	−	−	Serbia	[12]
*Cladosporium halotolerans*	1	+	+	+	−	Portugal, Germany	[15,18,23]
*Cladosporium herbarum*	1	+	+	+	−	Germany, Norway, Portugal, Serbia, UK	[9,11,12,15,20]
*Cladosporium macrocarpum*	1	−	+	−	−	Portugal	[18]
*Cladosporium oxysporum*	1	−	+	−	−	Serbia	[12]
*Cladosporium pseudocladosporioides*	1	−	−	+	−	Slovenia	[80]
*Cladosporium sphaerospermum*	1	−	+	+	−	Poland, Norway, UK	[9,20,28]
*Cladosporium* spp.	1	+	+	+	+	Greece, Slovakia, France, Austria, Portugal, Norway, Hungary, Belgium, Ukraine, Spain, UK	[5,7,8,9,10,13,14,15,16,17,18,81,82,83,84,87]
*Cladosporium tenuissimum*	1	−	+	−	−	Portugal	[15]
*Cladosporium variabile*	1	−	+	−	−	Serbia	[12]
*Clavispora lusitaniae*	1	−	−	+	−	Slovenia	[19]
*Clethridium corticola*	1	−	−	+	−	UK	[9]
*Clonostachys candelabrum*	1	−	+	+	−	Poland	[28]
*Coniochaeta hoffmannii*	1	+	+	+	−	Norway, Austria, Portugal	[14,18,20]
*Coniochaeta velutina*	1	−	+	−	−	Portugal	[18]
*Coniothyrium olivaceum*	1	−	+	+	−	UK	[9]
*Cordyceps bassiana*	1	+	−	+	−	Austria	[14]
*Cosmospora arxii*	1	+	−	+	−	Germany	[11]
*Cosmospora berkeleyana*	1	+	−	+	−	Germany	[11]
*Cosmospora butyri*	1	+	+	−	−	Norway	[20]
*Cosmospora* sp.	No data	−	+	−	−	Portugal	[15]
*Curvularia* spp.	1/2	+	−	+	−	Greece, Slovakia	[7,8]
*Cyclothyrium* sp.	No data	−	−	+	−	UK	[9]
*Cylindrocarpon* spp.	1/2	+	+	−	−	Slovakia, UK	[7,9]
*Cyphellophora europaea*	2	−	−	+	−	Germany	[23]
*Cyphellophora reptans*	1	+	−	+	−	Germany	[23]
*Cyphellophora sessilis*	1	+	−	+	−	Germany	[11,23]
*Cytospora* sp.	No data	−	+	+	−	UK	[9]
*Dactylaria* spp.	1/2	+	−	+	−	Slovakia, Austria	[7,14]
*Dactylella* spp.	1	+	−	+	−	Slovakia	[7]
*Debaryomyces hansenii*	1	−	−	+	+	Poland, Slovenia, France	[5,19,66]
*Didymella molleriana*	1	+	+	+	−	Norway, Austria, Portugal	[14,15,18,20]
*Didymella musae*	1	−	+	+	−	UK	[9]
*Diplocladium* spp.	No data	+	−	−	−	Slovakia	[7]
*Discosporium* sp.	No data	−	−	+	−	UK	[9]
*Doratomyces* spp.	1	−	−	+	−	Greece	[8]
*Embellisia* sp.	No data	−	+	−	−	UK	[9]
*Emmonsia* spp.	1/2	−	−	+	−	Greece	[8]
*Epicoccum nigrum*	1	+	+	+	−	Norway, Austria, UK, Serbia	[9,12,14,20]
*Epicoccum* spp.	1	−	−	+	−	Greece	[8]
*Eupenicillium* sp.	No data	−	−	+	−	UK	[9]
*Eurotium* spp.	1	−	−	+	−	Greece	[8]
*Exophiala alcalophila*	1	−	−	+	−	Slovenia, Germany	[19,23,67]
*Exophiala angulospora*	1	+	−	+	−	Germany	[11,23]
*Exophiala cancerae*	1	−	−	+	−	Germany	[23]
*Exophiala castellanii*	2	+	−	+	−	Germany, Poland	[11,23,66]
*Exophiala dermatitidis*	2	+	−	+	−	Slovenia	[19,67]
*Exophiala equina*	1	+	−	+	−	Germany	[23]
*Exophiala jeanselmei*	2	−	−	+	−	Poland, UK	[9,66]
*Exophiala lecanii-corni*	1	−	−	+	−	Slovenia, Germany	[19,23,67]
*Exophiala mesophila*	1	+	−	+	−	Slovenia, Germany	[19,23]
*Exophiala oligosperma*	2	+	−	+	−	Slovenia, Germany	[19,23]
*Exophiala opportunistica*	1	−	−	+	−	Germany	[23]
*Exophiala phaeomuriformis*	2	−	−	+	−	Slovenia, Germany	[19,23,67]
*Exophiala pisciphila*	1	+	−	+	−	Germany	[11]
*Exophiala psychrophila*	1	+	−	+	−	Germany	[23]
*Exophiala salmonis*	1	+	−	+	−	Germany	[23]
*Exophiala spinifera*	2	−	−	+	+	Poland	[66]
*Exophiala* spp.	1/2	+	−	+	−	Germany, Greece	[8,11]
*Exophiala xenobiotica*	1	+	−	+	−	Slovenia, Germany	[19,23]
*Fusarium begoniae*	1	−	+	−	−	Portugal	[15]
*Fusarium culmorum*	1	−	+	+	−	Serbia, UK	[9,12]
*Fusarium flocciferum*	1	−	+	−	−	UK	[9]
*Fusarium foetens*	1	−	+	−	−	Portugal	[18]
*Fusarium incarnatum*	1	−	+	−	−	Serbia	[12]
*Fusarium oxysporum*	2	+	+	+	−	Norway, Serbia, UK	[9,12,20]
*Fusarium solani*	2	+	+	+	−	Germany, Greece, Poland, Serbia, UK	[8,9,11,12,28]
*Fusarium sporotrichioides*	1	−	+	−	−	Serbia	[12]
*Fusarium* spp.	1/2	+	+	+	+	Germany, Slovakia, Austria, Portugal, Norway, Belgium, Ukraine, Spain, Hungary, UK	[7,9,10,11,14,15,16,17,18,79,81,84,87]
*Fusarium torulosum*	1	−	+	−	−	UK	[9]
*Fusicolla aquaeductuum*	1	−	−	+	−	UK	[9]
*Fusicolla merismoides*	1	+	−	+	−	Germany	[11]
*Galactomyces geotrichum*	1	−	+	+	−	Slovenia, Portugal, Poland, Serbia, UK	[9,12,18,19,28,67]
*Geomyces* sp.	No data	+	−	+	−	Germany	[11]
*Geotrichum* spp.	1/2	+	+	+	−	Slovakia, Norway, Hungary	[7,20,79]
*Gibberella avenacea*	1	−	+	+	−	UK	[9]
*Gibberella fujikuroi*	1	−	+	−	−	UK	[9]
*Gibberella gordonii*	1	−	+	−	−	Serbia	[12]
*Gibberella intricans*	1	−	+	−	−	UK	[9]
*Gliocladium* spp.	1	+	+	+	−	Greece, Slovakia, UK, Hungary	[7,8,9,79]
*Graphium silanum*	1	+	−	+	−	Austria	[14]
*Hormiscium* spp.	1/2	+	−	+	−	Slovakia	[7]
*Hyphopichia burtonii*	1	−	+	−	−	Portugal	[15]
*Humicola grisea*	1	−	−	+	−	Hungary	[79]
*Isaria farinosa*	1	+	+	+	−	Germany, Norway, Serbia	[11,12,20]
*Issatchenkia orientalis*	1	−	−	+	−	Poland	[66]
*Kloeckera* spp.	1	+	−	+	−	Greece, Portugal	[8,15]
*Kluyveromyces lactis*	1	−	−	+	−	Poland	[66]
*Kluyveromyces marxianus*	1	−	−	+	−	Poland	[66]
*Lecanicillium lecanii*	1	+	+	+	−	Germany, Poland, Norway	[11,20,28]
*Leptodontidium* sp.	No data	−	−	+	−	UK	[9]
*Leptosphaeria* sp.	No data	+	+	+	−	Austria, UK	[9,14]
*Leucostoma persoonii*	1	−	+	−	−	Norway	[20]
*Mauginiella* sp.	No data	−	−	+	−	UK	[9]
*Melanospora simplex*	1	−	+	+	−	Poland	[28]
*Metarhizium carneum*	1	+	+	−	−	Norway	[20]
*Meyerozyma caribbica*	1	−	−	+	−	Slovenia	[19,67]
*Meyerozyma guilliermondii*	1	−	−	+	−	Slovenia	[19]
*Microdochium* sp.	No data	+	−	+	−	Austria	[14]
*Microsphaeropsis* sp.	No data	−	+	−	−	UK	[9]
*Microsporum* spp.	1/2	−	−	+	−	Slovakia	[7]
*Monascus ruber*	1	−	+	−	−	Norway	[20]
*Monilia* spp.	1/2	+	−	+	−	Slovakia, Belgium	[7,16]
*Nakazawaea holstii*	1	−	+	−	−	Portugal	[15]
*Neurospora* sp.	No data	−	+	−	−	UK	[9]
*Ochroconis musae*	1	−	−	+	−	Germany	[23]
*Ochroconis* sp.	1	+	−	+	−	Germany	[11]
*Oosporidium margaritiferum*	1	−	−	+	−	Poland	[66]
*Paecilomyces* spp.	1	+	−	+	+	Slovakia, Austria, Norway, Belgium, Spain, Poland	[7,10,14,16,66,81]
*Paecilomyces variotii*	1	+	+	+	−	Norway, Austria, Greece	[8,14,20]
*Papulaspora* sp.	No data	+	−	+	−	Slovakia	[7]
*Paraconiothyrium* sp.	No data	−	+	−	−	Portugal	[15]
*Paraphaeosphaeria minitans*	1	−	+	−	−	Potugal	[18]
*Paraphaeosphaeria sporulosa*	1	−	+	−	−	Portugal	[15]
*Paraphoma fimeti*	1	+	−	+	−	Germany	[23]
*Paspalomyces* sp.	No data	+	−	+	−	Slovakia	[7]
*Penicillium atrofulvum*	1	−	+	−	−	Portugal	[18]
*Penicillium aurantiogriseum*	1	−	+	+	−	UK, Portugal	[9,15]
*Penicillium brevicompactum*	1	+	+	+	−	Germany, Norway, Portugal, UK	[9,11,13,18,20]
*Penicillium canescens*	1	−	+	−	−	Norway, Portugal, Serbia	[12,15,18,20]
*Penicillium chrysogenum*	1	+	+	+	+	Germany, Norway, Serbia, Slovenia, UK, Hungary	[9,11,12,20,80,84]
*Penicillium citrinum*	1	−	+	+	−	Norway, Portugal, UK	[9,15,18,20]
*Penicillium corylophilum*	1	+	+	+	−	Portugal, UK	[9,13,18]
*Penicillium dierckxii*	1	−	+	−	−	Portugal, Norway	[15,18,20]
*Penicillium digitatum*	1	−	+	−	−	Portugal	[18]
*Penicillium echinulatum*	1	−	+	−	−	UK	[9]
*Penicillium expansum*	1	−	+	+	−	Norway, Portugal, UK	[9,13,18,20]
*Penicillium glabrum*	1	+	+	+	+	Germany, Norway, Portugal, UK, France, Poland	[9,11,13,15,18,20,28,88]
*Penicillium griseofulvum*	1	−	+	+	−	Portugal, Serbia, UK	[9,12,13,15,18]
*Penicillium hirsutum*	1	−	−	+	−	UK	[9]
*Penicillium implicatum*	1	−	+	−	−	Norway, Portugal	[15,20]
*Penicillium janczewskii*	1	−	+	+	−	Norway, UK	[9,20]
*Penicillium jensenii*	1	−	+	−	−	Norway	[20]
*Penicillium lanosum*	1	−	+	−	−	Norway	[20]
*Penicillium megasporum*	1	−	+	−	−	Norway	[20]
*Penicillium melanoconidium*	1	−	+	−	−	Portugal	[15]
*Penicillium melinii*	1	−	+	−	−	Norway	[20]
*Penicillium miczynskii*	1	−	+	−	−	Norway	[20]
*Penicillium montanense*	1	+	+	−	−	Norway	[20]
*Penicillium novae-zeelandiae*	1	−	+	−	−	Portugal	[18]
*Penicillium ochrochloron*	1	−	+	−	−	Portugal	[15]
*Penicillium ochrosalmoneum*	1	−	−	+	−	UK	[9]
*Penicillium olsonii*	1	−	+	−	−	Norway, Portugal	[18,20]
*Penicillium oxalicum*	1	−	+	−	−	Norway	[20]
*Penicillium pancosmium*	1	−	+	−	−	Portugal	[18]
*Penicillium paxilli*	1	−	+	−	−	Norway	[20]
*Penicillium phoeniceum*	1	−	+	−	−	Norway	[20]
*Penicillium purpurogenum*	1	−	+	+	−	Norway, UK	[9,20]
*Penicillium raistrickii*	1	−	+	+	−	Norway, Portugal, UK	[9,13,15,20]
*Penicillium resedanum*	1	−	+	−	−	Serbia	[12]
*Penicillium restrictum*	1	−	+	−	−	Norway, Portugal	[15,18,20]
*Penicillium roseopurpureum*	1	−	+	−	−	Norway	[20]
*Penicillium sanguifluum*	1	−	+	−	−	Portugal	[18]
*Penicillium scabrosum*	1	−	+	−	−	Portugal	[15]
*Penicillium simplicissimum*	1	−	+	−	−	Norway, UK, Portugal	[9,18,20]
*Penicillium solitum*	1	−	+	+	−	Norway, UK, Portugal	[9,13,15,18,20]
*Penicillium spinulosum*	1	+	+	+	−	Norway, UK	[9,20]
*Penicillium* spp.	1/2	+	+	+	+	Germany, Greece, Slovakia, France, Austria, Norway, Belgium, Ukraine, Spain, Sweden, Portugal, Hungary	[5,6,7,8,10,11,14,16,17,79,81,87]
*Penicillium thomii*	1	−	+	−	−	Norway, Portugal, Serbia	[12,15,20]
*Penicillium verrucosum*	1	+	+	−	−	Norway, Serbia	[12,20]
*Penicillium virgatum*	1	−	+	−	−	Portugal	[18]
*Penicillium waksmanii*	1	−	+	+	−	Portugal, UK	[9,13]
*Penicillium westlingii*	1	−	+	−	−	Norway	[20]
*Phaeosphaeria juncophila*	1	+	−	+	−	Austria	[14]
*Phialemonium* sp.	No data	−	+	−	−	Portugal	[18]
*Phialocephala dimorphospora*	1	−	−	+	−	Germany	[23]
*Phialophora cyclaminis*	1	−	+	−	−	Norway	[20]
*Phialophora fastigiata*	1	+	+	+	−	Italy, Germany, Norway, UK	[9,20,23,89]
*Phialophora* spp.	1/2	+	−	+	−	Germany, Greece, Slovakia, Austria, Portugal, Sweden	[6,7,8,11,13,14]
*Phialophora verrucosa*	2	−	+	−	−	Norway	[20]
*Phoma herbarum*	1	+	+	+	−	Germany, Serbia	[11,12]
*Phoma leveillei*	1	+	+	+	−	Germany, Italy, UK	[9,11,89]
*Phoma macrostoma*	1	−	+	+	−	UK	[9]
*Phoma medicaginis*	1	−	+	+	−	Serbia, UK	[9,12]
*Phoma* sp.	No data	+	+	+	−	Poland, Norway, Portugal, Serbia	[10,12,15,20,28]
*Phomatodes nebulosa*	1	−	−	+	−	UK	[9]
*Phomopsis* spp.	1	+	−	+	−	Austria, UK	[9,14]
*Pichia fermentans*	1	−	−	+	−	Slovenia	[19]
*Pichia membranifaciens*	1	−	−	+	−	France, Greece	[5,8]
*Pilidium concavum*	1	+	+	+	−	UK, Portugal	[9,18]
*Priceomyces carsonii*	1	−	−	+	−	Poland	[66]
*Prosthecium pyriforme*	1	+	−	−	−	Portugal	[18]
*Pseudeurotium hygrophilum*	1	−	+	−	−	UK	[9]
*Pseudogymnoascus pannorum*	1	−	+	−	−	Norway	[20]
*Pseudogymnoascus roseus*	1	−	+	−	−	Norway	[20]
*Pseudopithomyces sacchari*	1	−	+	−	−	UK	[9]
*Purpureocillium lilacinum*	1	+	+	+	−	UK, Portugal, Poland, Norway, Italy	[9,18,20,28,89]
*Pyrenochaeta* spp.	1/2	−	+	+	−	Greece, Italy, UK	[8,9,89]
*Pyrenochaeta unguis-hominis*	2	−	−	+	−	Germany	[23]
*Rhinocladiella similis*	2	+	−	+	−	Slovenia, Germany	[19,23,67]
*Saccharomycopsis capsularis*	1	−	−	+	−	Poland	[66]
*Saprochaete suaveolens*	1	−	−	+	−	Poland	[66]
*Sarocladium kiliense*	2	−	+	+	−	Poland, UK	[9,66]
*Sarocladium strictum*	1	+	+	+	−	Germany, Italy, Norway, Serbia	[11,12,20,89]
*Sarocladium terricola*	1	−	+	+	−	Serbia, Poland	[12,28]
*Sclerotinia sclerotiorum*	1	−	−	+	−	Poland	[28]
*Scopulariopsis acremonium*	1	−	+	−	−	UK	[9]
*Scopulariopsis brevicaulis*	2	−	+	+	−	Greece, Norway, UK	[8,9,20]
*Scopulariopsis fusca*	1	−	+	+	−	Poland	[20,66]
*Scopulariopsis* spp.	1/2	−	−	+	−	Greece	[8]
*Sepedonium* spp.	1	−	−	+	−	Greece, Norway	[8,10]
*Sporothrix* spp.	1/2	−	+	+	−	UK	[9]
*Stachybotrys chartarum*	1	+	+	+	−	Poland, Portugal	[18,28]
*Stachybotrys* spp.	1	+	−	+	−	Greece, Slovakia	[7,8]
*Staphylotrichum* sp.	No data	−	+	−	−	Norway	[20]
*Stemphylium* sp.	No data	+	−	+	−	Slovakia	[7]
*Stephanoma strigosum*	1	−	−	+	−	Hungary	[79]
*Sydowia polyspora*	1	−	−	+	−	UK	[9]
*Talaromyces funiculosus*	1	−	+	−	−	Serbia	[12]
*Talaromyces minioluteus*	1	−	−	+	−	UK	[9]
*Talaromyces pinophilus*	1	−	−	+	−	UK	[9]
*Talaromyces ruber*	1	−	+	+	−	Poland	[28]
*Talaromyces rugulosus*	1	−	−	−	+	Poland	[66]
*Talaromyces verruculosus*	1	−	−	+	−	Slovenia	[67]
*Trichoderma asperellum*	1	−	+	−	−	Portugal	[18]
*Trichoderma citrinoviride*	1	−	+	+	−	Slovenia, Portugal	[18,80]
*Trichoderma harzianum*	1	+	+	+	−	Portugal, UK	[9,15,18]
*Trichoderma koningii*	1	−	+	+	−	Serbia, UK, Portugal	[9,12,18]
*Trichoderma longibrachiatum*	1	−	−	−	+	Poland	[66]
*Trichoderma pleuroticola*	1	−	+	−	−	Portugal	[18]
*Trichoderma polysporum*	1	−	+	+	−	UK	[9]
*Trichoderma pseudokoningii*	1	−	+	+	−	UK	[9]
*Trichoderma* spp.	1	+	+	+	−	Greece, Slovakia, Norway, France, Austria, Belgium, Spain, Serbia, Hungary	[5,7,8,10,12,14,16,20,79,81]
*Trichoderma viride*	1	+	+	+	−	Poland, Austria, Ukraine, Serbia	[12,14,17,28]
*Trichomonascus ciferrii*	1	−	−	+	−	Greece	[8]
*Trichothecium* sp.	No data	+	−	+	−	Greece, Slovakia, Hungary	[7,8,79]
*Trichophyton* sp.	No data	+	−	+	−	Slovakia	[7]
*Tritirachium* sp.	No data	+	−	+	−	Slovakia	[7]
*Truncatella angustata*	1	−	+	−	−	UK	[9]
*Varicosporium* spp.	1	+	−	−	−	Slovakia	[7]
*Verticillium* spp.	1	+	−	+	−	Greece, Slovakia, UK, Hungary	[7,8,9,79]
*Volutella* sp.	No data	+	−	+	−	Germany	[11]
*Westerdykella dispersa*	1	−	+	−	−	UK	[9]
*Wickerhamomyces anomalus*	1	−	−	+	−	Poland	[66]
*Yarrowia lipolytica*	1	−	−	+	−	Slovenia	[19]
**Basidiomycota (phylum)**							
*Apiotrichum montevideense*	1	−	−	+	−	Slovenia	[19,67]
*Cryptococcus* sp.	No data	−	+	−	−	Portugal	[15]
*Cystobasidiopsis lactophilus*	1	−	−	+	−	Poland	[66]
*Cystobasidium minuta*	1	−	+	+	−	France, Portugal	[5,15]
*Cystobasidium slooffiae*	1	−	−	+	−	Slovenia	[19,67]
*Cystofilobasidium lari-marini*	1	−	−	+	−	Poland	[66]
*Filobasidium magnum*	1	−	−	−	+	Norway	[86]
*Naganishia albida*	1	−	+	−	−	Portugal	[15]
*Rhizoctonia* spp.	1	+	−	−	−	Slovakia	[7]
*Rhodotorula glutinis*	1	−	+	+	−	France, Ukraine	[5,17]
*Rhodotorula mucilaginosa*	1	+	−	+	−	Slovenia	[19,67]
*Rhodotorula* spp.	1	+	+	+	−	Germany, Greece, Poland, Austria, Portugal	[8,11,14,15,66]
*Schizophyllum commune*	1	−	−	+	−	Slovenia	[67]
*Sporidiobolus salmonicolor*	1	+	−	−	−	Slovenia	[19]
*Sporobolomyces japonicus*	1	−	−	+	−	Poland	[66]
*Sporobolomyces ruberrimus*	1	−	−	+	−	Slovenia	[80]
*Sporotrichum* spp.	1/2	+	+	−	−	Slovakia, UK	[7,9]
*Stereum* sp.	No data	−	−	+	−	UK	[9]
*Tilletiopsis* sp.	No data	+	−	+	−	Germany	[11]
*Trametes versicolor*	1	+	−	+	−	Austria	[14]
*Trichosporon coremiiforme*	1	+	−	−	−	Slovenia	[19]
*Triodiomyces crassus*	1	−	−	+	−	Slovenia	[19,67]
**Mucoromycotina (subphylum)**							
*Absidia cylindrospora*	1	−	+	+	−	Norway, UK	[9,20]
*Absidia glauca*	1	−	+	+	−	Norway, UK	[9,20]
*Absidia* spp.	1/2	+	−	+	−	Slovakia, Spain	[7,81]
*Chaetocladium brefeldii*	1	−	+	−	−	UK	[9]
*Cunninghamella elegans*	1	−	+	−	−	Portugal	[18]
*Gongronella butleri*	1	−	+	−	−	UK	[9]
*Lichtheimia corymbifera*	2	−	+	−	−	Norway	[20]
*Mortierella alpina*	1	−	+	−	−	UK	[9]
*Mortierella elongata*	1	−	+	−	−	UK	[9]
*Mortierella zychae*	1	−	−	+	−	UK	[9]
*Mucor azygosporus*	1	−	+	−	−	Norway	[20]
*Mucor circinelloides*	1	−	+	+	−	Norway, UK	[9,20]
*Mucor fuscus*	1	−	+	−	−	UK	[9]
*Mucor hiemalis*	1	−	+	+	−	Norway, Serbia, UK	[9,12,20]
*Mucor moelleri*	1	−	+	−	−	UK, Portugal	[9,18]
*Mucor mucedo*	1	−	−	+	−	Greece	[8]
*Mucor plumbeus*	1	−	+	+	−	Norway, UK	[9,20]
*Mucor racemosus*	1	−	+	+	−	Portugal, UK	[9,15,18]
*Mucor* spp.	1/2	+	+	+	−	Germany, Slovakia, France, Norway, Spain, Serbia, Hungary	[5,7,10,11,12,18,79,81]
*Mucor strictus*	1	−	+	+	−	UK	[9]
*Rhizomucor* spp.	1/2	−	−	+	−	Norway	[10]
*Rhizopus arrhizus*	1	−	+	−	−	Ukraine	[17]
*Rhizopus* spp.	1/2	−	−	+	−	Greece, Slovakia, France, Norway, Spain	[5,7,8,10,81]
*Rhizopus stolonifer*	1	−	+	+	−	Portugal, UK, Serbia	[9,12,13]
*Syncephalastrum racemosum*	1	−	−	+	−	UK	[9]
*Umbelopsis isabellina*	1	−	+	−	−	UK	[9]
*Umbelopsis ramanniana*	1	−	+	+	−	UK	[9]

Legend: * BSL: Biosafety level; +: fungi were present in the water samples; −: fungi were absent from the water samples. Taxonomical data and data on Biosafety level were obtained from Centraalbureau voor Schimelcultures, Utrecht, The Netherlands (CBS), Index Fungorum and MycoBank databases.

**Table 2:** The list of the most common fungi isolated from different water sources in Europe, recognised as causative agents of opportunistic infections and other health effects on human health.

Fungal Species	Local or Systemic Infections	Allergenic Compounds	Mycotoxins Production	Irritative Compounds, MVOC, Odor	References
***Alternaria:*** *A. alternata*	respiratory infections, skin and nail infections, keratitis	X	X	No data	[32,145]
***Aspergillus:*** *A. flavus* *A. fumigatus* *A. niger* *A. terreus* *A. ustus* *A. versicolor*	disseminated infections, respiratory infections, subcutaneous infections, rhinocerebral infections, skin and nail infections, ear infections, keratitis	X	X	X	[32,146,147,148,149,150,151,152,153,154]
***Aureobasidium:*** *A. pullulans* *A. melanogenum*	skin and nail infections, keratitis	X	No data	No data	[32,155]
***Beauveria:*** *B. bassiana*	disseminated infections, keratitis	X	No data	No data	[32,156]
***Botrytis:*** *B. cinerea*	No data	X	No data	No data	[157]
***Candida:*** *C. albicans* *C. parapsilosis species complex*	disseminated infections, mucosal infections	X	No data	No data	[32,158,159]
***Chaetomium:*** *C. globosum*	respiratory infections, rhinocerebral infections, skin and nail infections	X	X	No data	[32,160]
***Cladosporium:*** *C. cladosporioides* *C. herbarum* *C. sphaerospermum*	respiratory infections, skin and nail infections, keratitis	X	No data	No data	[32,161,162,163]
***Epicoccum:*** *E. nigrum*	No data	X	No data	No data	[164]
***Exophiala:*** *E. dermatitidis* *E. jeanselmei*	disseminated infections, respiratory infections, skin and nail infections	No data	No data	No data	[32]
***Fusarium:*** *F. oxysporum* *F. solani*	disseminated infections, keratitis, skin and nail infections	X	X	No data	[32,165,166]
***Paecilomyces:*** *P. variotii*	disseminated infections, respiratory infections, keratitis, skin and nail infections	X	No data	No data	[32,167]
***Penicillium:*** *P. brevicompactum* *P. chrysogenum* *P. citrinum* *P. expansum* *P. glabrum* *P. simplicissimum*	respiratory infections, endocarditis, rhinocerebral infections, keratitis	X	X	X	[32,151,168,169,170,171,172]
***Purpureocillium:*** *P. lilacinum*	disseminated infections, respiratory infections, keratitis, subcutaneous infections, skin and nail infections	No data	No data	No data	[32]
***Sarocladium:*** *S. kiliense* *S. strictum*	disseminated infections, respiratory infections, keratitis, subcutaneous infections, skin and nail infections	No data	No data	No data	[32]
***Scopulariopsis:*** *S. brevicaulis*	skin and nail infections, keratitis, endocarditis	X	No data	No data	[32,173]
***Stachybotrys:*** *S. chartarum*	respiratory infections	X	X	No data	[174]
***Trichoderma:*** *T. harzianum* *T. viride*	disseminated infections, respiratory infections	X	X	X	[32,151,160,175]
***Rhodotorula:*** *R. mucilaginosa*	catheter-related fungemia	X	No data	No data	[32,176]
***Mucor:*** *M. circinelloides* *M. hiemalis* *M. racemosus*	disseminated infections, keratitis, rhinocerebral infections, skin and nail infections, subcutaneous infections	X	No data	No data	[32,177,178]
***Rhizopus:*** *R. arrhizus* *R. stolonifer*	disseminated infections, keratitis, subcutaneous infections, skin and nail infections	X	No data	No data	[32,179,180]

Legend: X; indicating the ability of fungi to produce allergenic compounds, mycotoxins, irritative compounds, MVOC and odor.
